# Antibiotic prescribing of village doctors for children under 15 years with upper respiratory tract infections in rural China: A qualitative study

**DOI:** 10.1097/MD.0000000000003803

**Published:** 2016-06-10

**Authors:** Zhixia Zhang, Xingxin Zhan, Hongjun Zhou, Fang Sun, Heng Zhang, Merrick Zwarenstein, Qian Liu, Yingxue Li, Weirong Yan

**Affiliations:** a Department of Epidemiology and Biostatistics, School of Public Health, Tongji Medical College, Huazhong University of Science and Technology, Wuhan; b Xianning Center for Disease Control and Prevention, Xianning, P.R. China; c Schulich School of Medicine & Dentistry Western University, Richmond, London, UK.

**Keywords:** antibiotic stewardship, primary health care, qualitative research

## Abstract

The aim of this study was to explore the knowledge, attitudes, and practices of village doctors regarding the prescribing of antibiotics for children under 15 years with upper respiratory tract infections (URTIs) in rural China. Twelve focus group discussions (FGDs) were conducted in Xianning, a prefecture-level city in rural China, during December 2014. We conducted 6 FGDs with 35 village doctors, 3 with 13 primary caregivers (11 parents), and 3 with 17 directors of township hospitals, county-level health bureaus, county-level Centers for Disease Control and Prevention, or county-level Chinese Food and Drug Administration offices. Audio records of the interviews were transcribed verbatim and analyzed using the thematic analysis approach. Participants believed that unnecessary antibiotic prescribing for children under 15 years with The occurrence of URTIs was a problem in village clinics in rural China. The discussions revealed that most of the village doctors had inadequate knowledge and misconceptions about antibiotic use, which was an important factor in the unnecessary prescribing. Village doctors and directors reported that the doctors’ fear of complications, the primary caregivers’ pressure for antibiotic treatment, and the financial considerations of patient retention were the main factors influencing the decision to prescribe antibiotics. Most of the primary caregivers insisted on antibiotics, even when the village doctors were reluctant to prescribe them, and they preferred to go to see those village doctors who prescribed antibiotics. The interviewees also gave their opinions on what would be the most effective measures for optimizing antibiotic prescriptions; these included educational/training campaigns, strict regulations on antibiotic prescription, and improved supervision. Findings emphasized the need to improve the dissemination of information and training/education, and implement legislation on the rational use of antibiotics. And it also provided helpful information to guide the design of more effective interventions to promote prudent antibiotic use and good antimicrobial stewardship.

## 1 Introduction

Antibiotic consumption is high and increasing in developing countries such as Thailand, India, South Africa, and China.^[1,2]^ About 85% to 90% of antibiotics are prescribed by primary care physicians, and upper respiratory tract infections (URTIs) such as pharyngitis, otitis media, common cold, rhinosinusitis, and bronchitis are the main reasons for antibiotic prescriptions.^[3,4]^ It was reported that nearly 50% of children were prescribed antibiotics for URTIs and that the prevalence rate in children under 15 years old was 3 times higher than in any other age group.^[5,6]^ However, most URTIs are of viral origin and do not require antibiotics for treatment.^[7]^ A systematic review in 2011 presented strong evidence that unnecessary use of antibiotics was one of the major causes of the emergence of antimicrobial resistance (AMR), which was a global public health threat, and spread in primary care settings.^[8]^ In low and middle-income countries, the unnecessary use of antibiotics among children is of special concern, because they are more vulnerable to invasive AMR,^[7]^ due to the higher prevalence of infectious diseases and the lower standards of sanitation and public health.^[9]^ The spread of AMR has led to increased rates of morbidity and mortality, and a high economic burden.^[10]^ To underscore this urgency, the World Health Organization chose “Antimicrobial Resistance: no action today, no cure tomorrow” as the theme of World Health Day in 2011.^[11]^ To standardize antibiotic utilization, the Chinese government also published “The Chinese Guidelines for the Clinical Application of Antimicrobial Agents in Acute Respiratory Infections”^[12]^ and “The Guidelines for the Use of Antibacterial Agents in Clinical Practice”.^[13]^

The behavior of prescribers plays a critical role in the consumption of antibiotics and interventions to improve this are a potential tool to curb the spread of AMR.^[14]^ In response to growing concern, knowledge about the driving forces behind antibiotic prescription is needed, through evaluation of prescribers’ knowledge, attitudes, and practices.^[14]^ Understanding this information will be helpful to develop policies and strategies to further optimize antibiotic prescription.

Researchers are increasingly focusing on the behavior of prescribers when prescribing antibiotics to children with URTIs.^[15–18]^ Previous studies suggested that prescribing behavior is complex^[19]^ and could be affected by multiple factors, such as doctors’ limited knowledge of prudent prescribing,^[20,21]^ a fear of complications,^[18,20,21]^ unfounded beliefs in the effectiveness of antibiotics,^[22]^ uncertainty of the diagnosis and management of URTIs,^[21,23]^ and parents’ expectations or pressure.^[21–23]^

China was the second largest consumer of antibiotics in the world between 2000 and 2010.^[2]^ It was reported that the overprescribing of antibiotics (up to 60%) was most common in village clinics^[24]^ and in pediatric clinical practice.^[1]^ The available studies in China were mostly focused on the antibiotic prescribing patterns, antibiotic utilization, and prescribing behaviors of physicians.^[6,11,25,26]^ Studies regarding the knowledge, attitudes, and practices of village doctors in antibiotic prescribing for children with URTIs in China in primary care settings are scarce.

Therefore, the aim of the study was to explore the key stakeholders’ perceptions of the village doctors’ knowledge, attitudes, and practices regarding antibiotic prescribing for children with URTIs, and to identify the factors associated with unnecessary antibiotic prescriptions.

## 2 Methods

### 2.1 Setting and participants

Focus group discussions (FGDs), a method used in qualitative exploration, was adopted to help explore the key stakeholders’ viewpoints through group interactions.^[20]^ We based our qualitative work on the grounded theory—an approach that derives theory from repeated analyses of data, used for understanding key stakeholders’ perceptions optimally.^[27,28]^

The key stakeholders included village doctors, primary caregivers, directors from the local county-level Centers for Disease Control and Prevention (CDC), Health Bureaus or China Food and Drug Administration (CFDA) offices, and township hospital staff who work in the field of drug administration and/or have direct contact with village doctors.^[18,29]^ A child, as defined by the United Nations Convention on the Rights of the Child,^[30]^ is “a human being below the age of 18 years.” However, antibiotic usage was the highest among children under 15 years,^[5]^ so “children” in the present study refers to “human beings under 15 years.”

This study was conducted in Xianning City, in the Hubei Province in central China, in December 2014. The gross domestic product (GDP) of the Hubei Province ranks ninth among the 31 provinces, autonomous regions, and municipalities of mainland China. The GDP of Xianning ranks ninth among the 17 prefecture-level cities in the Hubei Province. It is a prefecture-level city with approximately 2.48 million residents, 17.66% of which are children under 15 years.^[31]^ Three different rural counties from Xianning city (Chongyang, Jiayu, and Chibi) with different levels of economic development (low, medium, and high) were selected for this study. The GDP of Chibi ranks first among the 6 rural counties, and it had a population of 0.52 million, with 313 village doctors. Jiayu's GDP ranks second, with a population of 0.37 million, and 186 village doctors. Chongyang's GDP ranks fifth, with a population of 0.49 million, and 547 village doctors.^[32]^

Two towns were selected from each county based on their level of economic development (low and high) and proximity to the county center. In addition, in each selected town, 1 village close to the county center was selected, from which primary caregivers were chosen. Four FGDs were then held in each county; 2 groups consisted of village doctors from the 2 selected towns (1 FGD in each town), respectively, 1 group consisted of directors from the local public health/drug administration institutions (as mentioned above), and 1 group consisted of primary caregivers from the 2 selected villages. FGD participants were selected based on the following criteria: village doctors who currently provided medical care to children; directors in the drug administration field; primary caregivers of children who had visited a village doctor in the past 6 months with a URTI. To get a diverse range of perspectives on the topic and to maximize the variation of the sample group, interviewees were purposefully recruited from a wide range of demographic backgrounds (age, sex, and educational background). Village doctors and directors were approached with the support and assistance of the head director of the Xianning CDC. The primary caregivers were approached by the researchers, who searched the medical records in the selected village clinics and invited eligible participants by telephone. In total, 12 focus groups, each with 4 to 6 participants, were held. Theme saturation was reached by the end of the 12th FGD.

### 2.2 Interviews and data collection procedures

To ensure consistency across all the FGDs, and to minimize the influence of the interviewers’ professional backgrounds,^[33]^ all the FGDs were coordinated and conducted by the first (ZXZ) and second authors (XXZ). Both of them are healthcare researchers with a medical education background. They have been trained in qualitative interviewing as part of their doctoral (PhD) studies and have extensive experience in conducting interviews. ZXZ, who hosted all of the FGDs, was in charge of eliciting the participants’ thoughts on the interview topics. XXZ kept detailed handwritten notes of the nonverbal and verbal communication (quoted material) that emerged in the group discussions and might be used in the subsequent analyses.^[34]^ Audio recordings of the discussions were made with the participants’ consent. The discussions were conducted in Mandarin.

Semistructured interview guides were used to facilitate all of the FGDs. For the village doctors, the questions focused on their knowledge and beliefs about AMR, attitudes regarding the appropriate indications for antibiotics to treat URTIs, the number of times doctors prescribed antibiotics to children per week, and whether they had been trained in antibiotic prescription. For the directors, the interview guide included questions about their perceptions toward the village doctors’ knowledge, attitudes, and prescribing behavior. For the primary caregivers, the questions were about their expectations of and experiences with antibiotic treatment, whether they had asked the village doctors to prescribe antibiotics for their children, what condition their children had been in at the time, and their level of satisfaction with the visit to the doctor.

The FGDs with the village doctors (n = 6) occurred in a meeting room at each township's local hospital. The FGDs with the directors (n = 3) occurred at each county's local CDC, whereas the FGDs with primary caregivers (n = 3) occurred at their village clinics. Each FGD ranged from 50 to 90 minutes in length. All of the participants were compensated for transportation costs and their time, and there were no other incentives to participate in the FGDs.

### 2.3 Data analysis

The thematic analysis technique was employed.^[35]^ All of the discussions were transcribed verbatim and anonymized. Transcripts were reviewed against the audio recordings by the interviewers to ensure completeness and accuracy. The recurring viewpoints relevant to the focus group questions were listed and the interviewers individually went through the data to produce the initial codes line by line. As patterns emerged, these codes were categorized and then grouped into relevant themes.^[35]^ Subsequently, the interviewers discussed the emerging themes until a consensus on the overall themes was reached. The researchers used a constant comparative approach for moving back and forth between the data and emerging themes until all the data were analyzed.^[28]^ The analysis was performed using the NVivo 8 (QSR International, Melbourne, Australia) qualitative data analysis software.

### 2.4 Ethical considerations

Ethical approval for this study was provided by the Institutional Review Board of Tongji Medical College, Huazhong University of Science and Technology, China, on November 2014. Written consent was obtained before the study, and each participant was assured that their contribution was voluntary, individual comments were confidential, and not linked to any of the government authorities.

## 3 Results

A total of 65 participants took part in 12 focus groups. As presented in Table 1, a quarter (n = 17) were directors, 13 were primary caregivers, and 35 were village doctors. About a quarter (n = 4) of primary caregivers had 2 children, and average of their children aged 6.8 ± 3.9 years (mean ± SD).

**Table 1:**
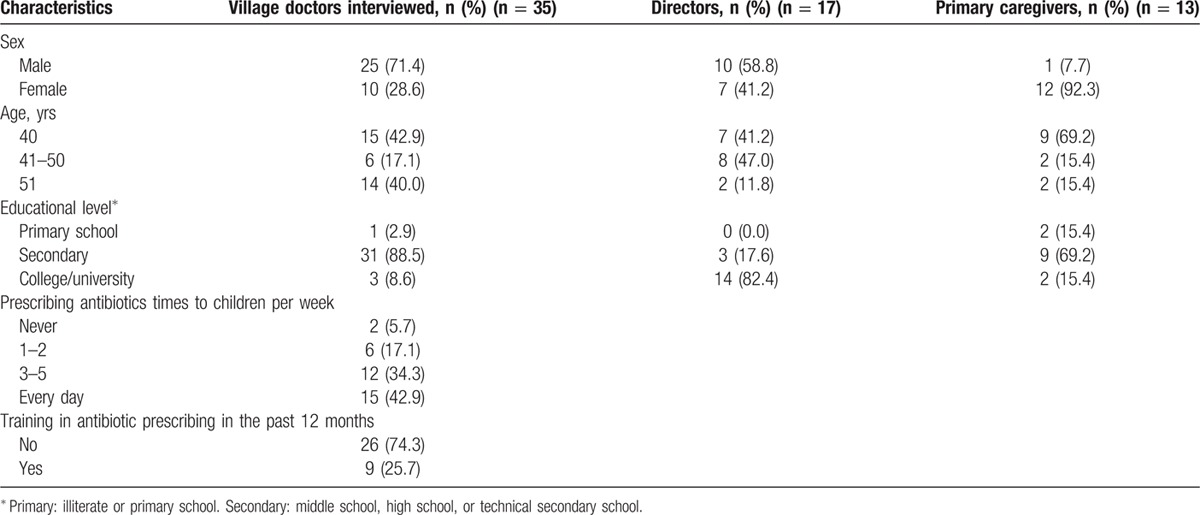
Participant sociodemographic characteristics.

Four overarching themes were identified. The first 3 themes emerged in line with the interview guides: knowledge, antibiotic prescription by village doctors, and attitudes toward antibiotic prescribing decisions. The last overarching theme, perceptions of potential interventions to improve antibiotics prescription emerged from the analysis. The third and fourth theme encompassed subthemes, and details were discussed below. Direct quotations of each interviewees which best illustrate the themes are presented in the texts. Only interviewees’ occupational roles and sexes are presented to maintain anonymity.

### 3.1 Knowledge

#### 3.1.1 Knowledge and awareness of AMR and its consequences

Almost all of the village doctors said that they had heard of the term “AMR.” However, few of them knew the accurate meaning of the term and the mechanism of AMR. Less than half (40.0%) demonstrated the correct knowledge of the consequences of AMR, such as treatment failure and possible future effects, on children seeking treatment for infectious disease.

“I have only heard of it (AMR), but I don’t know the exact meaning of AMR.” (Village doctor, female, FGD1).

“As to AMR, I am not sure whether my understanding is correct. For example, if you take the antibiotic which can kill the bacteria, but, gradually, this type of bacteria adapts to this antibiotic, it can no longer kill them. Is that it?” (Village doctor, male, FGD7).

Furthermore, the discussions showed that almost all of the interviewees (village doctors and directors) realized that AMR was not only a problem at all levels (community level and hospital level) in China, but also all over the world. One director mentioned that China was one of the heaviest antibiotic consumers in the world.

“Yes, AMR does exist, this is for sure. I think that AMR is a universal problem in our clinical practice.” (Village doctor, male, FGD 3).

“I think AMR is a huge problem. I definitely think that it is also a problem throughout the whole world. Besides, I feel that antibiotics consumption is heaviest in our country. For example, penicillin-resistance is common in medical care, and 88.24% of Gram-positive bacteria were reported to be resistant to it in our investigation in 2013.” (Director, male, FGD 10).

#### 3.1.2 Knowledge regarding the use of antibiotics for treatment of URTIs in children

Out of all the village doctors that we interviewed, 80.0% acknowledged that their knowledge of the use of antibiotics for treatment of URTIs was limited and only 8.6% said that they were confident about the optimal use of antibiotics. Only a few village doctors knew the appropriate ages for antibiotic use in children. Furthermore, the discussions demonstrated that three-fifths of the village doctors had misconceptions regarding the use of antibiotics for cough or runny nose with or without fever, and there was confusion regarding the role of antibiotics for viral infections. One common misconception was that antibiotics could shorten the duration and reduce the complications of URTIs. Moreover, 85.7% of the village doctors wished to be further educated about the appropriate use of antibiotics, including knowledge on the application and selection of antibiotics.

“I think that we are still lacking knowledge on antibiotics use. As you know, there are so many antibiotics now, it is difficult for us to select the appropriate antibiotics for children with URTIs.” (Village doctor, male, FGD3).

“I am not sure how to prescribe antibiotics to children with URTIs; under 3 years particularly. Both the choice of antibiotic type and the dose were too hard for me. If the patient were a child under 3 years, I would usually suggest to his/her parents to go to see the doctor in the township hospital or above.” (Village doctor, male, FGD6).

“Sometimes, I feel it is difficult to choose the correct dose and interval of antibiotics. And I am not sure whether I should prescribe antibiotics if the child had fever alone or when there is uncertainty in the diagnosis.” (Village doctor, female, FGD8).

The directors also commented that about three-fifths of village doctors aged 50 years and above had a poor educational background, so their knowledge was insufficient and their competence needed to be enhanced. Some directors explained that younger doctors were not willing to work in village clinics, because of the low salaries, poor working environment, and lack of promotion opportunities. When the primary caregivers were asked about their perceptions toward the village doctors’ competence, they responded that they were not quite clear about the village doctors’ medical skills. A small number of primary caregivers (23.1%) showed suspicion and distrust of the village doctor's competence.

“I think the competence of 85.0% of the village doctors needs to be enhanced, and most of them did not receive normal and professional training on antibiotics use.” (Director, male, FGD 5).

“80.0% of village doctors don’t know how to use antibiotics appropriately. For example, it is hard for them to differentiate when and how to use broad-spectrum antibiotics and narrow-spectrum antibiotics.” (Director, male, FGD 10).

“Most of the village doctors are 50 years old and with a low medical education background. I think their knowledge may not be enough.” (Primary caregiver, female, FGD 6).

### 3.2 Antibiotic prescription by village doctors

Almost all of the directors and primary caregivers thought that antibiotics were frequently prescribed in village clinics. The directors said that antibiotics were not only used incorrectly for susceptible bacterial infections, but also frequently prescribed for illnesses that were not caused by bacteria. The directors also stated that about one-third of village doctors would prescribe unnecessary antibiotics for children with URTIs. At the same time, 46.2% of the primary caregivers said that, most of the time, they do not know the reason for antibiotic prescription or the name of the drug being prescribed.

“According to the requirements of China's Ministry of Health, antibiotics prescription and consumption should be less than 20% for outpatients. But as we know, it was up to 80–90% (it is high) both in the township and village level.” (Director, male, FGD5).

“Anyway, they (village doctors) will prescribe you drugs when you visit. Most of the time, 90% of the prescriptions contain antibiotics.” (Parent, male, FGD 6).

As for the village doctors, when asked whether they prescribed antibiotics to children with URTIs, 82.9% of them commented that they would prescribe antibiotics. Furthermore, 11.4% of them felt that almost 100% of village doctors would prescribe antibiotics to children. On the other hand, only 11.4% of the village doctors reported that they refused to prescribe antibiotics unless pressured by the primary caregivers. Therefore, the attitudes toward antibiotic prescribing decisions were probed.

“Usually, we prescribe antibiotics to children for only 2 to 3 days; that is harmless for children.” (Village doctor, male, FGD12).

“100% of village doctors will use antibiotics, irrespective of the severity of URTIs (mild or severe).” (Village doctor, male, FGD3).

### 3.3 Attitudes toward antibiotic prescribing decisions

Almost all of the village doctors indicated that their antibiotic prescribing decisions were mainly guided by the children's symptoms. The symptoms for which antibiotics were frequently prescribed were fever (especially >38.5°), ranked first, followed by swollen tonsils and cough. Other reasons are discussed below.

#### 3.3.1 Fear of complications

The most frequently reported reason for prescribing antibiotics was a fear of complications, such as pneumonia, bronchitis, and otitis media. Most of the village doctors (74.3%) and directors (58.9%) said that since diagnostic tests, such as a routine blood test and a C-reactive test, were not readily available at village clinics in China, village doctors often face difficulties in differentiating between viral and bacterial infections, especially at the early stages of infection. To deal with this uncertainty, they made decisions based on their clinical experience and prescribed antibiotics to prevent complications.

“I think it's viral, but because of the lack of blood testing, no one knows if it may re-infect or turn out to be bacterial. In this case, we would prescribe antibiotics to them to prevent complications, and also maintaining the parents’ peace of mind.” (Village doctor, female, FGD7).

“Generally speaking, we think antiviral drugs are enough for a common cold. But if we use antibiotics, the antibiotics are helpful to reduce the complications, and the child will get better quickly.” (Village doctor, male, FGD3).

### 3.4 Primary caregivers’ pressure

The majority of the village doctors (85.7%) and directors (76.5%) commented that about 60.0% of primary caregivers gave their children multiple cycles of antibiotics through self-medication for 1 or 2 days before coming to see village doctors. Also, they want their children's illness alleviated as quickly as possible at the clinic. They demanded “strong” or “the best” medicines, and they usually requested for the antibiotics to be administered by intravenous infusion. Moreover, 25.6% of the village doctors reported that parents who had been previously prescribed antibiotics were more likely to ask for antibiotics again. Sometimes, the parents (usually the younger parents) named the antibiotics they wanted. To maintain a good doctor–patient relationship, about 70% of the village doctors complied with the primary caregivers’ request even when they felt the antibiotics were unnecessary.

“Most parents don’t know when and how to use antibiotics nor the consequences of AMR. Besides, they want an instant cure, so they ask village doctors to prescribe antibiotics.” (Director, male, FGD5).

“When they (the parents) come to the village clinic, most of them ask for the best medicine for their children. Sometimes, I think that antibiotics are unnecessary and I explain this to them. But they insist on their own opinions. Sometimes, they even quarrel with me. In this case, what else can I do? I can only prescribe antibiotics to them.” (Village doctor, male, FGD3).

In view of this, the primary caregivers were asked whether they would require antibiotics from the village doctors. The discussions showed that self-medication before going to see a village doctor was a common practice among most of them (69.2%) and that they did persist in requesting antibiotics after village doctors withheld a prescription for their children. Only 15.4% of them said that they thought the village doctors were the only ones who possessed good knowledge about drugs and that they followed the village doctors’ decisions.

“When my child gets a cough or running nose, I would buy over-the-counter (OTC) drugs first. If it doesn’t work after 2 or 3 days, then I go to see a doctor. I hope my child gets better as soon as possible, so I demand for the doctor to prescribe antibiotics by intravenous drips.” (Primary caregiver, female, FGD6).

### 3.5 Patient retention and financial considerations

Due to the pressure posed by primary caregivers, the village doctors feared that if they employed a wait-and-watch policy or prescribed only antiviral drugs for fevers or the common cold, the primary caregivers would not be satisfied and would go to other village doctors who would prescribe antibiotics. Furthermore, the primary caregivers would not return to the clinic the next time their child had a URTI.

“When a 5-year-old child with a running nose or slight cough visits, you can’t just say, well it is viral, you don’t need antibiotics, and you just need to drink more water. If you do not give the child anything, most of the parents think that you have a low level of knowledge and they would not come here the next time their children get URTIs.” (Director, male, FGD1).

“As we all know, the URTIs will self-recover in 5–7 days. But if you give antibiotics, the child will be better in 2–3 days. If I don’t prescribe antibiotics, the patient will visit another doctor who prescribes antibiotics. Then I have lost a patient.” (Village doctor, male, FGD3).

If their child's illness was not quickly treated at 1 village clinic, 76.9% of the primary caregivers would change physicians. Whereas 57.1% of the village doctors denied the existence of financial incentives to prescribe antibiotics (there is little profit from drug prescriptions since the implementation of the National Essential Medicine Policy [NEMP] in 2009^[36]^ and a new community-based rural health insurance scheme called the New Cooperative Medical Scheme [NCMS] in 2003),^[25]^ 41.2% of the directors commented that retention of patients would increase their consultation fees. This may be a reason for the high prevalence of antibiotic prescriptions.

“[The] Consultation fee is 5 RMB for each patient. If there are 10 patients in one day, that will be 50 RMB in total.”(Village doctor, male, FGD3).

### 3.6 Perceptions of potential interventions to improve antibiotics prescription

#### 3.6.1 Education or training for the prescribers

Almost all of the interviewees perceived educational or training sessions for physicians as the most effective method of improving the proper use of antibiotics. According to 60.0% of the village doctors, most of the training or education they received focused on disease diagnosis and care, instead of on the proper (rational) use of antibiotics. They suggested that regular updates on the rational use of antibiotics could enable them to make more appropriate decisions for individual patients. Additionally, 80.0% expressed a wish to have expert consultation on problematic cases, and continuous medical education.

“First, I think the most important thing is to educate the village doctors. It is necessary to develop training on the use of antibiotics and help them establish the proper attitude towards the use of antibiotics.” (Village doctor, male, FGD 6).

“I think there is a long way to go to improve antibiotic prescribing. The first and main thing is to strengthen their (prescribers) professional ability by conducting systematic training on the use of antibiotics. Although we (county-level CFDA) deliver some training every year, the quantity is not enough.” (Director, male, FGD 2).

“I feel that many village doctors have some difficulties in choosing the appropriate antibiotics for children. And enhancing their proficiency with the use of antibiotics is necessary.” (Primary caregiver, male, FGD 6).

#### 3.6.2 Educating the community

Tellingly, 88.2% of the directors and 91.4% of the village doctors emphasized that community education was crucial. They suggested that advertisements through newspapers, pamphlets, and local television were helpful to increase the understanding of the importance of the proper use of antibiotics.

“I think that population education is fundamental and is the key, as it is a good way to avoid pressure for antibiotic prescriptions, and helpful for maintaining a good doctor-patient relationship.” (Director, male, FGD5).

“What has to be done is to educate the population, so that they know what an antibiotic is, and the side effects of antibiotics. Once they get more knowledge on the use of antibiotics, they might not ask us for antibiotics.” (Village doctor, female, FGD1).

#### 3.6.3 Strict regulations and supervision of antibiotic prescribing

The FGDs revealed that existing authoritative guidelines on the use of antibiotics were readily available. But 58.8% of the directors and 34.3% of the village doctors commented that the existing regulations were lax and the directors also said that they faced some challenges when putting them into practice. There should be strict supervisory measures for antibiotic prescribing, such as strict routine checks and punishments for violations. For example, 1 director mentioned that physicians who unnecessarily prescribe antibiotics should receive a financial penalty.

“There are some guidelines on the usage of antibiotics in our clinical practice, such as “The guidelines for antibacterial use in clinical practice”, but it is hard to enforce them in village clinics, because the regulations could not effectively restrain their (village doctors) behavior.” (Village doctor, male, FGD3).

“We did have some guidelines in clinical practice, such as the Antibiotic Usage Guidelines of Hubei province in 2008. But we have some difficulties in implementing them. For example, there is poor supervision, and the relevant guidelines have not been effectively implemented at the township/village level, especially at the village level. So strengthening supervision is the key.” (Director, male, FGD10).

## 4 Discussion

Data revealed that antibiotics were commonly prescribed to children with URTIs in village clinics in rural China and the antibiotics prescription practices were shaped by multiple factors.

Although national guidelines regarding the optimal usage of antibiotics had already been published,^[12,13]^ previous studies reported that inappropriate antibiotic prescriptions in village clinics were worse than in other healthcare institutions, and the majority of antibiotic prescriptions in rural areas in China were for colds (78%) and acute bronchitis (93.5%).^[37,38]^ However, few studies focused specifically on antibiotic prescriptions to children with URTIs in village clinics in China. The findings in the present study complement the existing literature by providing the novel view that antibiotics were commonly prescribed for children with URTIs in rural China. This situation was similar to that in other developing countries, such as India, South Africa, Peru, and Vietnam.^[2,17]^ In these countries, antibiotics were used as substitutes for public health measures.^[2]^ It was reported that 58% of children with URTIs were given antibiotics when they went to see a doctor in rural Peru.^[4]^ Similar to previous studies, the greatest volume of antibiotic prescriptions in primary care settings were for fever, tonsillitis, and cough.^[15,20]^

A significant finding of this study that has not been reported in China is that the village doctors had an inadequate knowledge of the proper usage of antibiotics, which was an important factor in the rise of unnecessary antibiotic use. Worse than in rural Vietnam (27%), 60% of the Chinese village doctors did not fully understand the consequences of AMR.^[17]^ It is well known that antibiotics play a very limited role in the treatment of URTIs and show little benefit in preventing secondary complications or reducing the duration of URTIs.^[15]^ However, nearly half of the village doctors (48.6%) in this study overestimated the effectiveness of antibiotics, which was similar to primary pediatricians from Europe (43.5%) and Korea (70%).^[15,22]^ The main reason for their inadequate knowledge may be the lower educational background of most of the village doctors in rural China.^[29]^ Xu et al,^[39]^ who conducted a longitudinal study of the rural health workforce with 1927 village doctors in 5 counties in rural China, found that 91.2% of village doctors had a secondary education background or below and only 8.8% of them had a college/university education background. It was also suggested that the education/training regarding proper antibiotic usage in village clinics was insufficient.^[37]^ Because increasing the knowledge and awareness of antibiotics and AMR were key positive factors for promoting the appropriate use of antibiotics,^[40]^ efforts to increase the village doctors’ knowledge are strongly needed. The topics proposed in this study could be a target for future intervention.

Regarding the attitudes toward antibiotic prescribing, our findings were similar to that of a previous meta-synthesis.^[21]^ Uncertainty in the etiological diagnosis was reported as the main cause of fear when prescribing in primary care settings in other countries, such as Spain and India.^[20,21,41]^ In such instances, faced with fear of complications, prescribers tend to prescribe antibiotics, even when the indications may be questionable. Some measures were proposed to be helpful for eliminating the prescribers’ fear, such as access to rapid diagnostic tests, readily available clinical guidelines, and dissemination of knowledge regarding the proper use of antibiotics among the general population.^[20,21,23]^

Concerning the financial incentives for antibiotic prescribing, patient retention was proposed to be one of the main influencing factors, which was similar in primary care settings in other countries such as Korea and India.^[41,42]^ It was reported that the village doctors’ income mainly consisted of their salary, allowance for basic public health services, remuneration for drug sales, and a general fee for medical service since the implementation of healthcare reform policies in 2009.^[43]^ Since the introduction of the NEMP, all drugs on the National Essential Medicine List (NEML) are sold at zero-profit, meaning that the village doctors’ profits from drug sales are virtually nil.^[43]^ As a result, village doctors increasingly had to rely on salaries and subsidies from the government, such as the allowance for basic public health services.^[44]^ In addition, the village doctors thought that they only received a modest subsidy and had a modest income.^[29,45]^ Since the introduction of a fee-for-service policy in the 1990s, village doctors earned their living largely through fee-based medical services, and their income directly increases in proportion to their patient numbers.^[18,45]^ There is also evidence indicating that the prevalence of antibiotic prescriptions is higher when doctors are paid on a fee-for-service basis rather than a fixed salary.^[22]^

The percentage of primary caregivers who demanded or expected antibiotics was as high as 50% in some countries, and it was the second predictor for overprescribing.^[46,47]^ Failure to meet patients’ expectations was viewed as detrimental for doctor–patient relationships and posed a risk for losing patients to other doctors.^[21]^ Worse than another study conducted in rural areas of China, 70.0% of the village doctors in the present study said that they would prescribe antibiotics if their patients were insistent about getting them.^[37]^ A survey conducted in Korea reported that, when pressured by parents, nearly 80% of primary physicians would prescribe medicines and 40% would prescribe antibiotics, even if they thought the prescription was unnecessary.^[42]^ However, it was argued that the prescribers may have misunderstood or exaggerated the parents’ demands,^[19]^ and further study was needed to clarify this. Evidence has indicated that antibiotic prescription rates decrease when the general public is better informed on the proper usage of antibiotics.^[47]^ Therefore, this problem may be attributed to the insufficient knowledge of the primary caregivers in the present study.

To prevent further increases in antibiotic consumption, interventions that promote/encourage the appropriate use of antibiotics in developing countries should be a priority.^[2]^ Educational interventions should be targeted at prescribers, and also the general population. They should not only include knowledge about the rational use of antibiotics, but also emphasize the negative impact of overprescription and the importance of individual involvement in the prevention of AMR.^[11,21,47]^ Additionally, the development of tailored guidelines, feedback mechanisms for providers, strict regulations and supervision, and effective communication between prescribers and the community are beneficial for improving antibiotic use.^[1,23,47]^ It was found that some developed countries, such as France and the UK, had reduced antibiotic consumption through better prescription practices.^[48]^ Their successful experiences could provide models for developing countries. Keeping in mind the differences in economic and cultural contexts, policy makers and antibiotic stewards need to strike the right balance between curing patients at present and preserving the efficacy of antibiotics for future generations.^[2]^

### 4.1 Strengths and limitations

This study has several strengths. First, previous studies about antibiotics in village clinics mostly focused on the utilization of antibiotics (types and amounts), and antibiotic prescribing behaviors and patterns of the primary physicians. Also, most of the studies were conducted on a general population (outpatient/inpatient and adults) or for nonspecific disease.^[6,18,37,38]^ The current study is filling a gap by providing information on the village doctors’ knowledge, attitude, and practices for antibiotic prescription, specifically for children with URTIs. Second, it focused on the main topics of “Antibiotic Prescription” and “AMR,” which were emphasized by the WHO.^[49]^ Third, data triangulation between the key stakeholders maximized the ability to interpret the findings. Moreover, the current study provides baseline information for the design of future interventions for improving the appropriate prescribing of antibiotics in primary care settings in developing countries.

This study only included participants from Xianning City in China, so the generalization of the data is subject to limitations. However, data saturation was reached as no new themes were generated. Furthermore, to minimize any potential bias in representation, the interviewees had a large variety of socioeconomic characteristics. Informed by the findings of this study, a future study, which employed a quantitative methodology and a larger and nationally representative sample, might be considered to compare the attitudes of participants (village doctors, directors, and primary caregivers) with different characteristics (e.g., age and educational levels). Such a study could assess in greater detail whether the attitudes/knowledge were associated with the quality and quantity of village doctors’ antibiotic prescriptions.

## 5 Conclusions

This study is an important step toward a better understanding of the knowledge, attitudes, and practices of village doctors prescribing antibiotics to children under 15 years with URTIs in rural China in primary care settings. As evidenced in the data, unnecessary prescribing to children under 15 years with URTIs was a common problem in village clinics in rural China. It was associated with multiple factors, such as the poor knowledge of village doctors, patients’ demands, and financial incentives. Efforts to improve the quality of antibiotic prescribing require multisector cooperation, and long-term efforts should target the general population, and also prescribers. Examples of effective measures include health education or training on the proper use of antibiotics and powerful supervision. Further studies should focus on the development and implementation of such measures to promote prudent antibiotic use for children with URTIs.
